# 4-Bromo-2,6-dimethyl­aniline

**DOI:** 10.1107/S1600536807064641

**Published:** 2007-12-06

**Authors:** Rui Liu, Yu-Hao Li, Wei Luo, Shan Liu, Hong-Jun Zhu

**Affiliations:** a Department of Applied Chemistry, College of Science, Nanjing University of Technology, Nanjing 210009, People’s Republic of China

## Abstract

The asymmetric unit of the title compound, C_8_H_10_BrN, contains two independent mol­ecules. The Br, N and methyl group C atoms lie in the benzene ring planes. In the crystal structure, N—H⋯N hydrogen bonds link the mol­ecules.

## Related literature

For general background, see: Heravi *et al.* (2005 ▶). For bond-length data, see: Allen *et al.* (1987 ▶).
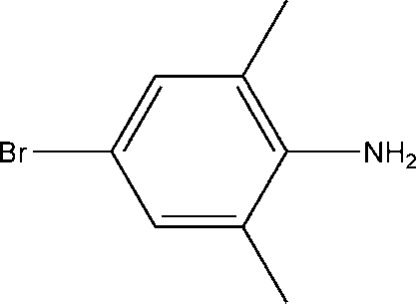


## Experimental

### 

#### Crystal data

C_8_H_10_BrN
*M* *_r_* = 200.07
Monoclinic, 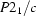

*a* = 20.141 (4) Å
*b* = 5.150 (1) Å
*c* = 17.300 (4) Å
β = 111.53 (3)°
*V* = 1669.3 (7) Å^3^
*Z* = 8
Mo *K*α radiation
μ = 4.85 mm^−1^
*T* = 294 (2) K
0.40 × 0.20 × 0.20 mm

#### Data collection

Enraf–Nonius CAD-4 diffractometer
Absorption correction: ψ scan (North *et al.*, 1968 ▶) *T*_min_ = 0.211, *T*_max_ = 0.379
3392 measured reflections
3268 independent reflections
1523 reflections with *I* > 2σ(*I*)
*R*_int_ = 0.040
3 standard reflections frequency: 120 min intensity decay: none

#### Refinement

*R*[*F* ^2^ > 2σ(*F* ^2^)] = 0.064
*wR*(*F* ^2^) = 0.166
*S* = 1.06
3268 reflections
183 parameters
H-atom parameters constrained
Δρ_max_ = 0.24 e Å^−3^
Δρ_min_ = −0.25 e Å^−3^

### 

Data collection: *CAD-4 Software* (Enraf–Nonius, 1985 ▶); cell refinement: *CAD-4 Software*; data reduction: *XCAD4* (Harms & Wocadlo, 1995 ▶); program(s) used to solve structure: *SHELXS97* (Sheldrick, 1997 ▶); program(s) used to refine structure: *SHELXL97* (Sheldrick, 1997 ▶); molecular graphics: *PLATON* (Spek, 2003 ▶); software used to prepare material for publication: *SHELXTL* (Bruker, 2000 ▶).

## Supplementary Material

Crystal structure: contains datablocks I, global. DOI: 10.1107/S1600536807064641/hk2405sup1.cif

Structure factors: contains datablocks I. DOI: 10.1107/S1600536807064641/hk2405Isup2.hkl

Additional supplementary materials:  crystallographic information; 3D view; checkCIF report

## Figures and Tables

**Table 1:** Hydrogen-bond geometry (Å, °)

*D*—H⋯*A*	*D*—H	H⋯*A*	*D*⋯*A*	*D*—H⋯*A*
N1—H1*A*⋯N2^i^	0.86	2.50	3.279 (10)	151
N2—H2*E*⋯N1^ii^	0.86	2.50	3.287 (10)	152

Symmetry codes: (i) 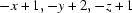; (ii) 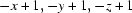.

**Fig. 1.**
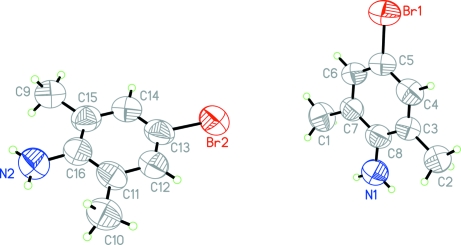
The molecular structure of the title molecule with the atom-numbering scheme. Displacement ellipsoids are drawn at the 50% probability level. Hydrogen bonds are shown as dashed lines.

**Fig. 2.**
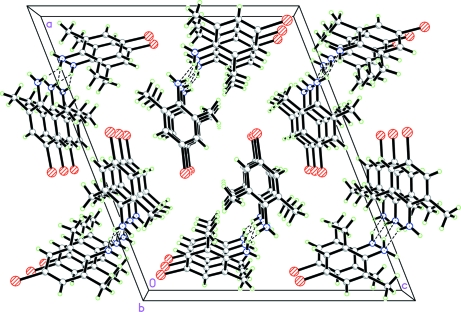
A packing diagram of (I). Hydrogen bonds are shown as dashed lines.

## supplementary crystallographic information

### Comment

The title compound, (I), contains amino and halogen groups, which can react with different groups to prepare various function organic compounds. It is a kind of aromatic organic intermediate that can be used for many fields such as aromatic conductive polymers and organometallic chemistry (Heravi *et al.*, 2005). We herein report its crystal structure.

The asymmetric unit of (I) contains two independent molecules (Fig. 1), in which the bond lengths and angles (Table 1) are within normal ranges (Allen *et al.*, 1987). The Br, N and C atoms of the methyl groups lie in the benzene ring planes.

In the crystal structure, intermolecular N—H···N hydrogen bonds (Table 2) link the molecules (Fig. 2), in which they may be effective in the stabilization of the structure.

### Experimental

The title compound, (I), was prepared by the literature method (Heravi *et al.*, 2005). The crystals were obtained by dissolving (I) (0.5 g) in hexane (20 ml) and evaporating the solvent slowly at room temperature for about 7 d.

### Refinement

H atoms were positioned geometrically, with N—H = 0.86 Å (for NH) and C—H = 0.93 and 0.96 Å for aromatic and methyl H, respectively, and constrained to ride on their parent atoms, with *U*_iso_(H) = *xU*_eq_(C), where *x* = 1.5 for methyl H, and *x* = 1.2 for all other H atoms.

### Crystal data

**Table:**

C_8_H_10_BrN	*F*_000_ = 800
*M**_r_* = 200.07	*D*_x_ = 1.592 Mg m^−^^3^
Monoclinic, *P*2_1_/*c*	Melting point: 321 K
Hall symbol: -P 2ybc	Mo *K*α radiation λ = 0.71073 Å
*a* = 20.141 (4) Å	Cell parameters from 25 reflections
*b* = 5.150 (1) Å	θ = 10–13º
*c* = 17.300 (4) Å	µ = 4.85 mm^−^^1^
β = 111.53 (3)º	*T* = 294 (2) K
*V* = 1669.3 (7) Å^3^	Needle, colorless
*Z* = 8	0.40 × 0.20 × 0.20 mm

### Data collection

**Table:**

Enraf–Nonius CAD-4 diffractometer	*R*_int_ = 0.040
Radiation source: fine-focus sealed tube	θ_max_ = 26.0º
Monochromator: graphite	θ_min_ = 1.1º
*T* = 294(2) K	*h* = −24→23
ω/2θ scans	*k* = 0→6
Absorption correction: ψ scan(North *et al.*, 1968)	*l* = 0→21
*T*_min_ = 0.211, *T*_max_ = 0.379	3 standard reflections
3392 measured reflections	every 120 min
3268 independent reflections	intensity decay: none
1523 reflections with *I* > 2σ(*I*)	

### Refinement

**Table:**

Refinement on *F*^2^	Secondary atom site location: difference Fourier map
Least-squares matrix: full	Hydrogen site location: inferred from neighbouring sites
*R*[*F*^2^ > 2σ(*F*^2^)] = 0.064	H-atom parameters constrained
*wR*(*F*^2^) = 0.166	*w* = 1/[σ^2^(*F*_o_^2^) + (0.050*P*)^2^ + 0.6*P*] where *P* = (*F*_o_^2^ + 2*F*_c_^2^)/3
*S* = 1.06	(Δ/σ)_max_ = 0.002
3268 reflections	Δρ_max_ = 0.24 e Å^−^^3^
183 parameters	Δρ_min_ = −0.25 e Å^−^^3^
Primary atom site location: structure-invariant direct methods	Extinction correction: none

### Special details

**Table:**

Geometry. All e.s.d.'s (except the e.s.d. in the dihedral angle between two l.s. planes) are estimated using the full covariance matrix. The cell e.s.d.'s are taken into account individually in the estimation of e.s.d.'s in distances, angles and torsion angles; correlations between e.s.d.'s in cell parameters are only used when they are defined by crystal symmetry. An approximate (isotropic) treatment of cell e.s.d.'s is used for estimating e.s.d.'s involving l.s. planes.
Refinement. Refinement of *F*^2^ against ALL reflections. The weighted *R*-factor *wR* and goodness of fit *S* are based on *F*^2^, conventional *R*-factors *R* are based on *F*, with *F* set to zero for negative *F*^2^. The threshold expression of *F*^2^ > σ(*F*^2^) is used only for calculating *R*-factors(gt) *etc*. and is not relevant to the choice of reflections for refinement. *R*-factors based on *F*^2^ are statistically about twice as large as those based on *F*, and *R*- factors based on ALL data will be even larger.

### Fractional atomic coordinates and isotropic or equivalent isotropic displacement parameters (Å^2^)

**Table:**

	*x*	*y*	*z*	*U*_iso_*/*U*_eq_	
Br1	0.08550 (5)	0.9358 (3)	0.10030 (5)	0.1246 (5)	
Br2	0.43509 (5)	0.5936 (2)	0.34568 (6)	0.1157 (4)	
N1	0.1751 (3)	1.3344 (13)	0.4526 (4)	0.100 (2)	
H1A	0.2092	1.4455	0.4708	0.120*	
H1B	0.1557	1.2726	0.4854	0.120*	
N2	0.7343 (3)	0.1666 (14)	0.4436 (4)	0.102 (2)	
H2D	0.7635	0.2300	0.4226	0.123*	
H2E	0.7482	0.0463	0.4805	0.123*	
C1	0.2375 (4)	1.5677 (16)	0.3431 (5)	0.103 (3)	
H1C	0.2540	1.6065	0.2989	0.154*	
H1D	0.2771	1.5124	0.3912	0.154*	
H1E	0.2165	1.7202	0.3565	0.154*	
C2	0.0684 (4)	0.9407 (18)	0.4006 (5)	0.103 (3)	
H2A	0.0354	0.8059	0.3727	0.154*	
H2B	0.0437	1.0737	0.4183	0.154*	
H2C	0.1056	0.8692	0.4482	0.154*	
C3	0.1002 (4)	1.0549 (18)	0.3425 (5)	0.086 (2)	
C4	0.0812 (4)	0.9643 (17)	0.2630 (5)	0.090 (2)	
H4A	0.0475	0.8324	0.2448	0.108*	
C5	0.1113 (4)	1.066 (2)	0.2090 (5)	0.095 (2)	
C6	0.1609 (4)	1.2610 (17)	0.2362 (4)	0.086 (2)	
H6A	0.1803	1.3325	0.1998	0.103*	
C7	0.1822 (4)	1.3524 (15)	0.3159 (5)	0.079 (2)	
C8	0.1512 (4)	1.2540 (18)	0.3696 (5)	0.086 (2)	
C9	0.6901 (4)	0.5486 (19)	0.3171 (5)	0.108 (3)	
H9A	0.7332	0.6128	0.3586	0.163*	
H9B	0.6673	0.6858	0.2790	0.163*	
H9C	0.7013	0.4084	0.2874	0.163*	
C10	0.6417 (4)	−0.0549 (16)	0.5187 (5)	0.101 (3)	
H10A	0.6562	−0.2025	0.4948	0.152*	
H10B	0.6024	−0.1023	0.5346	0.152*	
H10C	0.6810	0.0023	0.5669	0.152*	
C11	0.6190 (4)	0.1636 (15)	0.4553 (5)	0.083 (2)	
C12	0.5507 (4)	0.2581 (19)	0.4319 (5)	0.093 (2)	
H12A	0.5193	0.1906	0.4550	0.112*	
C13	0.5290 (4)	0.4543 (18)	0.3736 (5)	0.089 (2)	
C14	0.5726 (4)	0.5468 (19)	0.3354 (5)	0.097 (3)	
H14A	0.5561	0.6727	0.2942	0.116*	
C15	0.6406 (5)	0.4529 (17)	0.3581 (5)	0.087 (2)	
C16	0.6641 (4)	0.2596 (18)	0.4174 (5)	0.087 (2)	

### Atomic displacement parameters (Å^2^)

**Table:**

	*U*^11^	*U*^22^	*U*^33^	*U*^12^	*U*^13^	*U*^23^
Br1	0.1232 (8)	0.1683 (11)	0.0770 (6)	−0.0228 (7)	0.0304 (5)	−0.0229 (6)
Br2	0.0927 (6)	0.1499 (9)	0.0949 (7)	0.0165 (6)	0.0231 (5)	0.0050 (7)
N1	0.096 (4)	0.113 (6)	0.087 (5)	−0.010 (4)	0.029 (4)	−0.010 (4)
N2	0.092 (4)	0.116 (6)	0.095 (5)	0.001 (4)	0.028 (4)	−0.003 (4)
C1	0.098 (6)	0.088 (6)	0.117 (7)	−0.006 (5)	0.033 (5)	−0.009 (6)
C2	0.093 (5)	0.119 (7)	0.094 (6)	−0.004 (5)	0.032 (5)	0.013 (6)
C3	0.077 (4)	0.106 (6)	0.070 (5)	0.002 (5)	0.023 (4)	0.006 (5)
C4	0.084 (5)	0.091 (6)	0.085 (5)	0.005 (4)	0.020 (4)	−0.002 (5)
C5	0.092 (5)	0.126 (7)	0.063 (4)	−0.007 (5)	0.024 (4)	0.002 (5)
C6	0.086 (5)	0.105 (6)	0.065 (5)	−0.007 (5)	0.026 (4)	0.008 (5)
C7	0.073 (4)	0.085 (5)	0.074 (4)	−0.003 (4)	0.021 (3)	0.005 (4)
C8	0.082 (4)	0.095 (5)	0.073 (4)	0.007 (4)	0.020 (4)	0.002 (4)
C9	0.105 (6)	0.136 (8)	0.083 (5)	0.006 (6)	0.033 (5)	0.000 (6)
C10	0.109 (6)	0.091 (6)	0.092 (6)	−0.002 (5)	0.023 (5)	−0.008 (5)
C11	0.080 (5)	0.078 (6)	0.076 (5)	−0.009 (4)	0.009 (4)	−0.008 (4)
C12	0.084 (5)	0.109 (7)	0.077 (5)	−0.003 (5)	0.019 (4)	0.002 (5)
C13	0.090 (5)	0.104 (7)	0.062 (4)	0.014 (5)	0.013 (4)	−0.013 (5)
C14	0.095 (6)	0.120 (7)	0.071 (5)	0.001 (5)	0.025 (4)	0.006 (5)
C15	0.094 (5)	0.095 (6)	0.071 (5)	−0.009 (5)	0.029 (4)	−0.010 (5)
C16	0.079 (5)	0.104 (6)	0.075 (5)	−0.009 (5)	0.023 (4)	−0.014 (5)

### Geometric parameters (Å, °)

**Table:**

Br1—C5	1.880 (8)	Br2—C13	1.913 (8)
N1—C8	1.399 (9)	N2—C16	1.403 (9)
N1—H1A	0.8600	N2—H2D	0.8600
N1—H1B	0.8600	N2—H2E	0.8600
C1—C7	1.520 (10)	C9—C15	1.503 (11)
C1—H1C	0.9600	C9—H9A	0.9600
C1—H1D	0.9600	C9—H9B	0.9600
C1—H1E	0.9600	C9—H9C	0.9600
C2—C3	1.497 (10)	C10—C11	1.520 (10)
C2—H2A	0.9600	C10—H10A	0.9600
C2—H2B	0.9600	C10—H10B	0.9600
C2—H2C	0.9600	C10—H10C	0.9600
C3—C4	1.367 (10)	C11—C12	1.373 (10)
C3—C8	1.405 (11)	C11—C16	1.391 (10)
C4—C5	1.390 (11)	C12—C13	1.380 (11)
C4—H4A	0.9300	C12—H12A	0.9300
C5—C6	1.374 (11)	C13—C14	1.363 (11)
C6—C7	1.369 (10)	C14—C15	1.368 (10)
C6—H6A	0.9300	C14—H14A	0.9300
C7—C8	1.390 (10)	C15—C16	1.382 (11)
			
C8—N1—H1A	120.0	C16—N2—H2D	120.0
C8—N1—H1B	120.0	C16—N2—H2E	120.0
H1A—N1—H1B	120.0	H2D—N2—H2E	120.0
C7—C1—H1C	109.5	C15—C9—H9A	109.5
C7—C1—H1D	109.5	C15—C9—H9B	109.5
H1C—C1—H1D	109.5	H9A—C9—H9B	109.5
C7—C1—H1E	109.5	C15—C9—H9C	109.5
H1C—C1—H1E	109.5	H9A—C9—H9C	109.5
H1D—C1—H1E	109.5	H9B—C9—H9C	109.5
C3—C2—H2A	109.5	C11—C10—H10A	109.5
C3—C2—H2B	109.5	C11—C10—H10B	109.5
H2A—C2—H2B	109.5	H10A—C10—H10B	109.5
C3—C2—H2C	109.5	C11—C10—H10C	109.5
H2A—C2—H2C	109.5	H10A—C10—H10C	109.5
H2B—C2—H2C	109.5	H10B—C10—H10C	109.5
C4—C3—C8	119.0 (7)	C12—C11—C16	119.4 (8)
C4—C3—C2	120.7 (8)	C12—C11—C10	118.6 (8)
C8—C3—C2	120.3 (7)	C16—C11—C10	121.8 (8)
C3—C4—C5	121.2 (8)	C11—C12—C13	119.3 (8)
C3—C4—H4A	119.4	C11—C12—H12A	120.4
C5—C4—H4A	119.4	C13—C12—H12A	120.4
C6—C5—C4	119.1 (8)	C14—C13—C12	121.5 (8)
C6—C5—Br1	120.2 (6)	C14—C13—Br2	119.9 (7)
C4—C5—Br1	120.7 (7)	C12—C13—Br2	118.5 (7)
C7—C6—C5	121.1 (7)	C13—C14—C15	119.5 (8)
C7—C6—H6A	119.5	C13—C14—H14A	120.3
C5—C6—H6A	119.5	C15—C14—H14A	120.3
C6—C7—C8	119.8 (7)	C14—C15—C16	120.1 (8)
C6—C7—C1	119.1 (7)	C14—C15—C9	121.1 (8)
C8—C7—C1	121.1 (7)	C16—C15—C9	118.8 (8)
C7—C8—N1	120.6 (8)	C15—C16—C11	120.1 (8)
C7—C8—C3	119.7 (7)	C15—C16—N2	121.0 (8)
N1—C8—C3	119.5 (7)	C11—C16—N2	118.9 (8)
			
C8—C3—C4—C5	0.0 (12)	C16—C11—C12—C13	−2.7 (12)
C2—C3—C4—C5	−178.9 (8)	C10—C11—C12—C13	−179.3 (7)
C3—C4—C5—C6	−0.2 (13)	C11—C12—C13—C14	3.7 (13)
C3—C4—C5—Br1	179.1 (6)	C11—C12—C13—Br2	−177.2 (6)
C4—C5—C6—C7	1.6 (13)	C12—C13—C14—C15	−3.4 (13)
Br1—C5—C6—C7	−177.8 (6)	Br2—C13—C14—C15	177.5 (6)
C5—C6—C7—C8	−2.6 (12)	C13—C14—C15—C16	2.2 (13)
C5—C6—C7—C1	179.6 (8)	C13—C14—C15—C9	179.6 (8)
C6—C7—C8—N1	176.4 (7)	C14—C15—C16—C11	−1.2 (12)
C1—C7—C8—N1	−5.9 (12)	C9—C15—C16—C11	−178.7 (7)
C6—C7—C8—C3	2.3 (12)	C14—C15—C16—N2	−177.9 (7)
C1—C7—C8—C3	−180.0 (7)	C9—C15—C16—N2	4.6 (12)
C4—C3—C8—C7	−1.0 (12)	C12—C11—C16—C15	1.5 (12)
C2—C3—C8—C7	177.8 (7)	C10—C11—C16—C15	178.0 (7)
C4—C3—C8—N1	−175.1 (7)	C12—C11—C16—N2	178.2 (7)
C2—C3—C8—N1	3.7 (12)	C10—C11—C16—N2	−5.3 (11)

### Hydrogen-bond geometry (Å, °)

**Table:**

*D*—H···*A*	*D*—H	H···*A*	*D*···*A*	*D*—H···*A*
N1—H1A···N2^i^	0.86	2.50	3.279 (10)	151
N2—H2E···N1^ii^	0.86	2.50	3.287 (10)	152

Symmetry codes: (i) −*x*+1, −*y*+2, −*z*+1; (ii) −*x*+1, −*y*+1, −*z*+1.
